# Do fair value measurements affect accounting-based earnings quality? A literature review with a focus on corporate governance as moderator

**DOI:** 10.1007/s11573-020-01025-6

**Published:** 2021-02-04

**Authors:** Johannes Thesing, Patrick Velte

**Affiliations:** Faculty of Business and Economics, Institute of Management, Accounting and Finance (IMAF), Leuphana University Lueneburg, Universitaetsallee 1, 21335 Lueneburg, Germany

**Keywords:** Fair value, Corporate governance, Audit, Earnings quality, Earnings management, G32, G41, M41, M42, M48

## Abstract

This structured literature review of 48 archival-based studies investigates the influence of fair value measurements on earnings quality and stresses the moderating impact of corporate governance. We focus on accounting-based earnings quality measures that have several advantages for investigating agency-related earnings management behavior compared to market-based measures (e.g. value relevance studies). Fair value measurements are not restricted to specific industries, periods, circumstances, or items in our sample. Based on the applied earnings quality measure, the reviewed articles are structured into five categories: (1) earnings persistence and predictive ability, (2) discretionary accruals, (3) target beating and properties of analysts’ forecasts, (4) earnings variability, and (5) other earnings quality measures. We indicate three key findings: first, fair value measurements show mixed earnings quality; second, lower-level fair value measurements decrease earnings quality; and third, corporate governance measures enhance earnings quality. After that, we deduce six research questions for future research. We show possible extensions to previous research designs in methodology and settings. Future research should also focus on corporate governance variables to a greater extent, especially compensation and board structures. Thereby, we suggest extending the neoclassical view with behavioral aspects.

## Introduction

Managerial discretion in fair value accounting gives rise to agency-related problems and creates signalling opportunities (Landsman 2007), and it is thereby subject to extensive discussion. Proponents mention that managerial discretion can be used informatively and enhances the relevance of financial reporting (Barth 2018; Beaver and Venkatachalam 2003). On the other hand, opponents argue that managers exploit discretion in fair value measurements and thus decrease the reliability of financial reporting (Shalev et al. 2013; Hitz 2007),^1^ a situation which may be influenced by corporate governance (Shleifer and Vishny 1997). Besides researchers, regulators also discuss whether managerial discretion affects the decision usefulness of fair value measurements. A recent example is the IASB’s post-implementation review of IFRS 13, which acknowledges that managerial discretion remains challenging in practical application (IASB 2018). While researchers and regulators primarily question the decision usefulness of fair valued accounting items, investors and analysts are primary interested in whether fair value measurements contribute to the overall assessment of management and business operations (Georgiou 2018). Since the exploitation of fair value measurements can impair or enhance the decision usefulness of fair value measurements, as well as performance evaluation, empirical evidence is informative for researchers, regulators, and practitioners.

We investigate whether managers use fair value measurements for earnings management, which may be defined as using judgement or structuring transactions for information or contracting purposes (Healy and Wahlen 1999). Earnings management and decision usefulness are empirically investigated with earnings quality measures (Dechow et al. 2010a; Dechow and Schrand 2004), which can be partitioned in *market-based* earnings quality measures, such as value-relevance studies, and *accounting-based* earnings quality measures (Francis et al. 2004). We focus on accounting-based earnings quality measures because they, unlike market-based measures, provide insights into fair value-related accruals management, which are more direct (Francis et al. 2004; Bernard 1993; Aboody et al. 1999), can be interpreted without additional assumptions (Dechow and Schrand 2004; Dechow et al. 2010a), and provide more homogeneous results.

Fair value accounting is subject to extensive research and literature reviews. The dominant research designs are value relevance studies (Barth et al. 2001; Holthausen and Watts 2001). Besides value relevance, samples of financial industries, some of which focus on the financial crisis, are dominant in fair value accounting research (e.g. Lobo 2017; Beatty and Liao 2014; Laux 2012). Other reviews consider articles that investigate fair value accounting issues and fair valued items using different methodologies (e.g. Sellhorn and Stier 2019; Campbell et al. 2019; Filip et al. 2017). Hairston and Brooks (2019) review the relation between financial reporting quality, including market-based measures, and accounting for derivatives. We cannot find any review that investigates managerial discretion in fair value measurements, regardless of specific standards, using mainly financial reporting data.

Thus, we focus on accounting-based earnings quality measures, inspired by Francis et al. (2004), and we do not restrict the sample to specific industries, periods, circumstances, or items. We review archival-based studies for reasons of comparability, which appears to be the most dominant methodology among earnings quality studies. We further contribute to previous research because we explicitly show whether corporate governance, such as board characteristics, influences potential fair value-related earnings management. We hold the incorporation of corporate governance as essential for three reasons. First, corporate governance mechanisms are a tool to mitigate opportunistic behavior (Shleifer and Vishny 1997). Second, corporate governance research may guide future regulatory efforts that have gained attention over the last decades (e.g. Obermann 2020; Kovermann and Velte 2019; Gerum et al. 2018). Third, research history shows that two research fields, corporate governance research and accounting research, converge and several accounting topics cannot be interpreted appropriately without considering corporate governance implications (Brown et al. 2011; Armstrong et al. 2010).

We summarize 48 studies according to the following accounting-based earnings quality measures: (1) earnings persistence and predictive ability, (2) discretionary accruals, (3) target beating and properties of analysts’ forecasts, (4) earnings variability, and (5) other earnings quality measures. Our literature review indicates three key findings. First, fair value measurements provide mixed results. Second, lower-level fair value measurements decrease earnings quality. Third, stronger corporate governance enhances earnings quality. We consider further limitations and recommendations for future research. First, we show extensions and improvements to earnings quality research in methodology and settings. Previous earnings quality research designs can be improved via incorporating managerial incentives, textual analyses, ex post analyses, and experiments. We emphasize the need for strong theory and exploitation of unique settings to mitigate endogeneity concerns. Thereby, future research may exploit current and recent macroeconomic shocks as well as regulatory shocks to governmental regulations and fair value regimes. We also highlight investment properties as a specific suitable setting for fair value-related accounting-based earnings quality research. Second, future studies should incorporate a greater variety of corporate governance mechanisms, especially regarding compensation and board structures. To align managerial behavior, we also recommend expanding the common perceptions of neoclassical principal-agent theory by incorporating behavioral issues.

Our literature review is structured as follows. Section 2 briefly describes the neoclassical principal-agent theory as the dominant theoretical framework and emphasizes signalling issues. Section 3 explains the selection of the reviewed studies (Sect. 3.1) and illustrates earnings quality (Sect. 3.2.1) and corporate governance measures (Sect. 3.2.2). Section 4 summarizes the reviewed articles, and Sect. 5 shows the limitations of the current research and gives recommendations to expand future research.

## Theoretical framework

Fair value measurements rely heavily on managerial assumptions and require managerial discretion (Marra 2016; Hilton and O’Brien 2009; Fargher and Zhang 2014). This results in information asymmetries between managers (agents) and investors (principals) (Landsman 2007). Therefore, fair value accounting gives rise to moral hazard (Landsman 2007) if we consider the additional assumptions of conflicts of interests between both groups and utility-maximizing participants (Jensen and Meckling 1976; Arrow 1985). Managers may exploit fair value measurements opportunistically and thereby decrease the reliability of information (Ramanna 2008; Ramanna and Watts 2012; Yao et al. 2018), which we refer to as adverse earnings management. Consequently, we assume that neoclassical principal-agent theory (Jensen and Meckling 1976; Fama and Jensen 1983; Arrow 1985) serves as the dominant theoretical framework to investigate earnings management in fair value accounting.

In the context of these agency risks, corporate governance serves to mitigate agency conflicts (Shleifer and Vishny 1997). According to Jain and Jamali (2016), we partition (corporate) governance mechanisms into four levels: (1) *institutional* relates to the environment rather than the organisation directly, (2) *firm* (e.g. ownership structure), (3) *group* (e.g. board structure and compensation), and (4) *individual* (e.g. CEO characteristics). We add external auditors to firm level corporate governance mechanisms, who face a particular role in agency conflicts because they assure the reasonableness of fair value measurements (ISA 540).

Under information asymmetry, managers can convey private information by making credible decisions, that is, by *signalling* (Leland and Pyle 1977; Ross 1977). Besides real business decisions, discretionary accounting-related decisions, such as forecasting, providing voluntary information, or disclosure, generally can be used for signalling if this information enables financial statement users to judge a high quality of information (Morris 1987; Healy and Palepu 1993; Landsman 2007). Therefore, managers may use discretion in fair value accounting to provide private information credibly and thereby enhance the relevance of information (Barth 2018; Beaver and Venkatachalam 2003), which we refer to as beneficial earnings management.

## Data selection and empirical framework

### Data selection

According to our theoretical framework, managers can exploit discretion in fair value measurements, either adversely or beneficially, and corporate governance may affect this behavior. To shed some light on these theoretical considerations and to structure previous evidence, we analyze related empirical findings via a structured literature review, inspired by Massaro et al. (2016).^2^ The data selection is based on the term *fair value* in connection with six (groups of) keywords for obtaining an objectified sample. We used the terms *discretion* to refer to the origin of managerial behavior and *earnings management* to refer to managerial behavior that can be explained by adverse or beneficial earnings management. Furthermore, we used the term *corporate governance* to refer to mechanisms that affect managerial behavior. We also used the terms *audit*, *auditing*, and *auditor* to refer to auditing issues as a specific set of corporate governance.

We collected articles from six major academic databases: *Google Scholar*, *ScienceDirect*, *JSTOR*, *ISI Web of Science*, *Wiley Online Library*, and *Scopus*. To limit the findings from the extensive fair value literature to the relevant articles, we applied the groups of keywords on the search metrics *title*, *abstract*, and *keywords*, depending on whether the databases allow one or all three of these metrics. We began the data collection in August 2018 and last updated it in July 2019. According to that methodology, we collected 514 unique articles. To ensure scientific quality of our sources, we excluded 49 working papers and 171 articles that ranked below the lowest category of the journal rankings for *ABS Guide 2018*, *ABDC 2016*, and *VHB JOURQUAL 3*, which include qualitative characteristics in their ranking methodology.^3^ After that, we excluded 96 articles that lacked sufficient empirical methodologies. Additionally, we dropped 36 articles because their abstract suggests that their content insufficiently relates to fair value accounting or relates to mathematical issues regarding estimating fair values.

Through our search metrics and standardized selection of studies, we obtained a sample of 162 potentially relevant articles that cover discretion, earnings management, and corporate governance (including auditing) topics in connection with fair value accounting. This procedure enabled us to select an empirical framework objectively that best fits the investigation of earnings management in fair value accounting and shows how corporate governance influences this relation. We selected earnings quality research designs because they represent the most prominent setting in which to investigate earnings management (e.g. Burgstahler and Dichev 1997; Healy and Wahlen 1999; Dechow et al. 2010a).^4^ In order to obtain comparable results, we focus on archival-based studies, which appear to be the most dominant methodology among earnings quality studies. We differentiate between two groups of earnings quality measures^5^: accounting-based and market-based (Francis et al. 2004). While accounting-based measures solely investigate accounting information, market-based measures incorporate market prices and returns of firms, that is, market assessments. Examples for the letter are value relevance (Barth et al. 2001; Holthausen and Watts 2001; Beatty and Liao 2014) and conditional conservatism (Kim et al. 2013; Badia et al. 2017; Black et al. 2018).

Based on the theoretical framework and among the variety of earnings quality measures, we decide to summarize indicators that are largely accounting-based for several closely related reasons. First, according to agency theory, we are interested in accruals management via fair value measurements, which would violate or strengthen the adequacy cash flow periodization. Accounting-based earnings quality proxies provide guidance on this, whereas market-based earnings quality proxies relate to whether earnings mirror economic income (Francis et al. 2004). Second, accounting-based earnings quality proxies enable a more direct investigation of the intended relation without considering market participants’ perceptions and related risk of additional confounding factors (Bernard 1993; Aboody et al. 1999; Francis et al. 2004). Third, although interpreting accounting-based earnings quality proxies also have their challenges, researchers do not need to consider additional assumptions as it is the case for interpreting market-based earnings quality proxies, such as those regarding the market efficiency (Dechow and Schrand 2004; Dechow et al. 2010a). Fourth, excluding studies with market-based earnings quality measures provide more homogenous results.

Applying this methodology, we obtained 29 out of 162 studies. Additionally, we reviewed references of the first sections of these articles for snowball sampling and identified a further 19 studies that fit the previously mentioned methodology. Consequently, the final sample of this structured literature review consists of 48 articles. We provide an overview of these studies in Table 1.

**Table 1:** Summary of reviewed papers

Panel A: by publication year		
2019: 2	2014: 6	2009: 2
2018: 4	2013: 3	2006: 1
2017: 6	2012: 3	2003: 1
2016: 4	2011: 4	1999: 2
2015: 7	2010: 1	1995: 2
Panel B: by sample region		
Australia: 2	Denmark: 1	Russia: 1
Brazil: 1	France: 2	Transnational: 6
Canada: 1	Malaysia: 2	UK: 2
China: 1	Portugal: 1	USA: 28
Panel C: by journal		
Accounting and Business Research: 1	British Accounting Review: 1	Journal of Applied Accounting Research: 1
Accounting and Finance: 1	Contemporary Accounting Research: 4	Journal of Banking and Finance: 1
Accounting Forum: 1	Corporate Ownership and Control: 3	Journal of Business Finance and Accounting: 3
Accounting Horizons: 2	International Journal of Disclosure and Governance: 1	Journal of Contemporary Accounting and Economics: 1
Accounting Review: 6	Journal of Accounting and Economics: 1	Journal of International Accounting Research: 2
Advances in Accounting: 4	Journal of Accounting and Public Policy: 1	Journal of International Accounting, Auditing and Taxation: 1
Asia–Pacific Journal of Accounting and Economics: 1	Journal of Accounting Research: 2	Research in Accounting Regulation: 1
Australian Journal of Management: 1	Journal of Accounting, Auditing and Finance: 2	Review of Accounting Studies: 6

Investigating the influence of fair value measurements on accounting-based earnings quality measures in connection with moderating corporate governance contributes to the previous research in three ways. First, market-based earnings quality measures are incorporated or exclusively used in the reviews of Barth et al. (2001), Holthausen and Watts (2001), and Hairston and Brooks (2019). Second, we do not focus on specific industries or periods, such as financial industries or the financial crisis (Beatty and Liao 2014; Laux 2012). Third, we do not focus on specific issues or items that are related to fair value accounting, such as long-lived operating assets (Sellhorn and Stier 2019) or derivatives (Hairston and Brooks 2019; Campbell et al. 2019). Derivatives account only for a small fraction in our final sample. A larger share of derivative studies may not support the quality of our results. On the one hand, these items are not always subject to extensive fair value accounting (Choi et al. 2015; Barton 2001). On the other hand, the association between earnings quality measures and derivatives, especially in the case of hedging, is very special (Campbell 2015; Choi et al. 2015; Makar et al. 2013). Table 2 gives an overview of the major fair value-related accounting standards.

**Table 2:** Institutional background of major fair value regulation of US-GAAP and IFRS

Topic	Standard(s)	Content extract
Financial instruments	ASC 320, SFAS 115 (superseded), IFRS 9, and IAS 39 (superseded)	Major categories of financial instruments: * Held-to-maturity*/*Amortised cost* (at amortized cost), * Loans and receivables* (at amortized costs) (only IAS 39), * Available-for-sale*/*Fair value through other comprehensive income* (at fair value with unrealized gains and losses included in other comprehensive income [OCI]; reclassification if required), and * Trading*/*Fair value through profit or loss* (at fair value with unrealized gains and losses included in earnings);
Financial instruments disclosure	ASC 825, SFAS 107 (superseded), and IFRS 7	Firms shall disclose certain information on financial instruments, e.g. regarding fair value measurements
Fair value option	ASC 825, SFAS 159 (superseded), IFRS 9, and IAS 39 (superseded)	Firms may apply fair value measurement for certain financial instruments/items
Hedging	ASC 815, SFAS 133 (superseded), IFRS 9, and IAS 39 (almost superseded)	In case of designation and qualification for hedging, subsequent adjustments to the fair value of the hedging instruments are recognized according to the hedging relationship that result from effective hedge proportions: * Fair value hedges* (gains and losses are included in earnings generally), * Cash flow hedges* (unrealized gains and losses are included in OCI; reclassification if required), and * Net investment hedges/in a foreign operation* (unrealized gains and losses are included in OCI; reclassification if required)
Goodwill	ASC 350, SFAS 142 (superseded), IFRS 3, and IAS 36	Goodwill is subject to impairment testing instead of amortizing. In case of impairment, the carrying amount is higher than the (implied) fair value (US-GAAP) or the recoverable amount (IFRS)
Revaluation model	IAS 16, and IAS 38	Firms may subsequently measure property, plant, and equipment as well as intangible assets at fair value less than accumulated impairment losses and accumulated depreciation and amortization
Investment property	IAS 40	Firms may measure all investment property subsequently at fair value or at cost. Subsequent measurement at cost requires disclosure of fair values
Fair value measurement	ASC 820, SFAS 157 (superseded), and IFRS 13	For fair value accounting in general, these standards mandate definitions, a measurement framework, and disclosure requirements, such as those regarding levels of fair value measurements in the fair value hierarchy

### Empirical framework

#### Earnings quality research

**Fig. 1:**
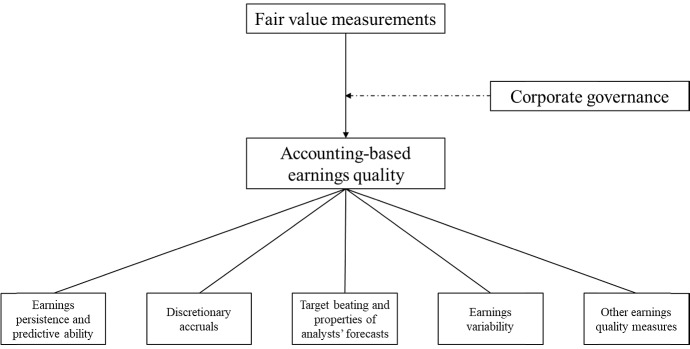
Research framework

Inspired by Francis et al. (2004), we review studies with accounting-based earnings quality measures as the output (dependent) variables. Related to suggestions from the earnings quality literature (e.g. Francis et al. 2004; Dechow et al. 2010a), we partitioned studies in the final sample according to the earnings quality measures in five categories: (1) *earnings persistence and predictive ability*, (2) *discretionary accruals*, (3) *target beating and properties of analysts’ forecasts*, (4) *earnings variability*, and (5) *other earnings quality measures*. Additionally, we review the moderating influence of corporate governance on the relation between fair value measurements and earnings quality. Figure 1 provides an overview over the research framework.

If appropriate, we provide some insights into whether earnings management is conducted via *real* or *accrual-based* actions. In the limitations, we criticize the studies using diverging assumptions regarding attributing earnings management to real or accrual-based actions, if the studies address the differentiation between real- and accrual-based earnings management at all. Generally, while managers use real business decisions and transactions for real earnings management, they achieve accrual-based earnings management via the accounting treatment of given decisions and transactions (Lev and Kunitzky 1974; Healy and Wahlen 1999; Roychowdhury 2006).

##### Earnings persistence and predictive ability

The literature uses the degree of *earnings persistence* as a measure of earnings quality (Francis et al. 2004; Dechow et al. 2010a). A higher degree of earnings persistence is assumed to be more decision useful for equity valuation (Dechow et al. 2010a). This ability of financial reporting information to support users in predicting future earnings is also an integral part of the relevance-objective of international standard setters (IFRS Conceptual Framework 2010/2018; Evans et al. 2014; Lee 2011; Bratten et al. 2016). In earnings persistence research designs, future earnings are regressed on aggregated current earnings, cash flows, and accruals, and on financial statement components, but other information, such as input (independent) variables, is also common in recent literature (Dechow et al. 2010a). *Predictive ability* is also of particular importance in fair value research and is closely related to earnings persistence. Fair value measurements and adjustments to them are expected to reflect the present value of estimated future cash flows (Barth 2014; Bratten et al. 2016) and adjustments to these estimations, respectively (Bandyopadhyay et al. 2017). This relation is assumed to be enhanced if managers use discretion in fair value measurements to signal information (Beaver and Venkatachalam 2003; Bandyopadhyay et al. 2017). If managers exploit discretion in fair value measurements opportunistically, the proposed relation may decrease (Aboody et al. 1999; Filip et al. 2015; Bandyopadhyay et al. 2017). Future cash flow as an output variable may also indicate real earnings management. We interpret a positive association of fair value measurements and related earnings, with future profitability measurements as less biased and of higher quality, with lower opportunism—in other words, desirable. We review studies that use fair value measurements as input variables, either on the balance sheet or on the statement of comprehensive income. We stick to the term *predictive ability,* but when we refer to *earnings persistence*, we explicitly point to an input variable that is a fair value-related, flow-sized earnings item.

##### Discretionary accruals

Researchers frequently use the residual component of accruals (*discretionary accruals*) as a measure of accrual-based earnings management and earnings quality (Dechow et al. 2010a; Jones 1991). Generally, an increase (decrease) of discretionary accruals may indicate a lower (higher) degree of earnings quality and a higher (lower) degree of opportunism.

##### Target beating and properties of analysts’ forecasts

Earnings that slightly meet or beat certain targets are important for earnings quality research. Firms more frequently report a gain than a loss, especially when earnings are close to zero (Burgstahler and Dichev 1997; Dechow et al. 2010a). Since firms try to turn small losses into gains, slightly positive earnings may indicate opportunistically managed earnings and lower earnings quality (Dechow et al. 2010a). The same interpretation holds for firms that report earnings that slightly beat consensus analysts’ forecasts (Degeorge et al. 1999; Payne and Robb 2000; Dechow et al. 2010a). Although measures of analysts’ forecasts accuracy and dispersion depend on perceptions of parties outside the firm, we consider few related studies in this review because they are closely linked to target beating measures.

##### Earnings variability

*Earnings variability* is another earnings quality measure, but the literature interprets it in connection with the accounting-based earnings attributes of smoothness and accrual quality (Francis et al. 2004; Dechow and Dichev 2002; Leuz et al. 2003; Lang et al. 2006). The interpretation of earnings variability is found to be twofold (Dechow and Schrand 2004): considering smoothness, lower earnings variability may indicate a higher degree of earnings management (Kohlbeck and Warfield 2010; Leuz et al. 2003).^6^ Otherwise, lower earnings variability can indicate a higher degree of accrual and earnings quality (Dechow and Dichev 2002). Researchers measure the variability as a standard deviation or variance of some part of the earnings or their residuals (e.g. Francis et al. 2004; Barth et al. 2008; Kohlbeck and Warfield 2010). We stick to the term variability regardless of the term used in the reviewed studies. We try to consider if the associations with earnings variability indicate higher or lower earnings quality.

#### Corporate governance

Significant governance mechanisms at the institutional level are the character of legal rules and the quality of law enforcement that proxy for investor protection (La Porta et al. 1997, 1998). However, there are also other institutional governance indicators as according to Kaufmann et al. (2009). Unlike authors who use external scores to construct their variables, some authors use corporate governance scores to measure corporate governance quality, although these aggregated measures need to be treated cautiously (e.g. Bhagat et al. 2008). Regarding firm level corporate governance mechanisms, some studies also use the characteristics of external auditors as a corporate governance moderator. Therefore, auditor size, specialization, and independence may contribute to higher earnings quality (Lin and Hwang 2010). The Big N audit firms are treated as large auditors (Lin and Hwang 2010) or directly as an overall indicator of audit quality (Becker et al. 1998). Another fair value-related corporate governance mechanism is the source of fair value measurements. External appraisers are found to provide more reliable fair value estimates than internal appraisers (e.g. Dietrich et al. 2001) and are therefore suspected to enhance earnings quality. The literature regarding our definition of group level corporate governance mechanisms suggests a positive relation between earnings quality and board independence, as well as board expertise, especially related to audit committees (e.g. Lin and Hwang 2010).

## Influence of fair value measurements on earnings quality and moderating corporate governance

### Earnings persistence and predictive ability

Overall, fair value measurements or related adjustments of fixed asset revaluations (Aboody et al. 1999), investment properties (Israeli 2015), and financial instruments (Beaver and Venkatachalam 2003; Dong et al. 2014; Evans et al. 2014) show a positive relation with future profitability. The literature stresses three methodical issues regarding the predictive ability of financial fair value measurements. First, fair value adjustments from revaluations of financial assets may predict net income only when they are coded binary instead of metric (Goncharov and van Triest 2011) in opposition to additional results of Aboody et al. (1999). Second, Evans et al. (2014) emphasize that studies obtain less biased results when they use future earnings from the investigated items as an output variable rather than earnings in general, which may be correlated with other factors besides the favoured association (Sloan 1999). Third, the predictive ability seems to depend on the discretionary classification of financial instruments. Among the different classifications, the fair less historical cost value (Park et al. 1999), adjustments (Evans et al. 2014),^7^ and net unrealized gains and losses (Bratten et al. 2016) of available-for-sale securities may positively predict earnings. Only Xie (2016) found a negative predictive ability of available-for-sale securities for resulting unrealized gains and losses. However, the results of Xie (2016) might not be attributed to decreasing earnings quality because available-for-sale fair value measurements do not appear procyclical. Negative associations are more prevalent among the predictive ability of unrealized gains and losses on cash flow hedges for future profitability (Bratten et al. 2016; Makar et al. 2013; Campbell 2015). Regarding the prediction of future credit losses, fair values may also be a bad predictor compared to net historical cost (Cantrell et al. 2014).

Goodwill write-offs, according to SFAS 142, show some predictive ability for future operating cash flow (Jarva 2009). Compared to pre-SFAS 142 periods, this relation and also the predictive ability of the balance sheet item seem to increase (Lee 2011). However, goodwill-related, acquired in-process research and development (R&D) does not seem to evoke meaningful differences in predictive ability and earnings management after they need to be fair valued instead of expensed (Chung et al. 2019). Goodwill impairment postponing suspects also show mixed results in SFAS 142 regimes: there are neither indications for opportunism nor for exceeding signalling (Lee 2011), indications for conveying information rather than for opportunism (Jarva 2009), or slight indications for opportunism (Filip et al. 2015). Shaari et al. (2017) investigated impairment reversals’ (excluding goodwill) predictive ability under IAS 36, and their findings support the previous indications that firms do not exploit impairments of fair value measurements opportunistically. However, the findings show some support for opportunism among earnings management suspects (Shaari et al. 2017).

We expect that managers exploit discretion on disclosed and recognized information differently (Schipper 2007). Managers may signal information through disclosed items and may exploit recognized items for opportunistic reasons (Beaver and Venkatachalam 2003), but we cannot find support for this hypothesis. Studies investigating the predictive ability of disclosed (e.g. Beaver and Venkatachalam 2003) or recognized (e.g. Dong et al. 2014) fair value measurements find similar results. Incorporating both types of information suggests the equal relevance of fair value measurements for future profitability (Israeli 2015).

Higher proportions of more reliably measured financial instruments seem to have greater predictive ability for future performance (Bratten et al. 2016). Different levels of fair value measurements also serve as a proxy for differences in reliability (Yao et al. 2018; Lin et al. 2017; Landsman 2007). He et al. (2018) do not find the predictive ability of unrealized adjustments to Level 3 fair value measurements. However, Evans et al. (2014) find lower earnings quality. Along the levels of fair value hierarchy (from Level 1 to Level 3), the earnings of lower-level fair value measurements show decreasing earnings persistence but the predictive ability of fair valued assets seems to increase in economical and statistical significance (Yao et al. 2018). However, the authors do not provide interpretations of these at least partially contradicting results. Altamuro and Zhang (2013) find lower-level fair value measurements can enhance one-quarter-ahead cash flow persistence, but they also find overall mixed results for the differences between Level 2 and Level 3 fair value measurements. Proxying for all future cash flows, Altamuro and Zhang (2013) indicate that managerial discretion in fair value measurements can be beneficial.

Lower-level fair values also tend to enhance conservative accounting behavior and conditional conservatism may enhance lower-level fair value measurements’ predictive ability (Black et al. 2018). The predictive ability of investment properties’ fair value adjustments for quarterly future cash flow seems to grow with the term length but is statistically significant for every term length when the firms yield lower accruals, which may be the result of corporate governance and constraining lending contracts (Bandyopadhyay et al. 2017). Similarly, Lopes and Walker (2012) initially found a negative association between fixed asset revaluations and the future operating income of Brazilian firms, but this opportunistic behavior mitigates with higher scores on a local corporate governance index. Yao et al. (2018) provide further evidence that stronger enforcement, audit environment, and auditor industry expertise overall strengthen the persistence of lower-level fair value-related earnings. Similarly, there are indications that audit committee expertise and scrutiny of Big 4 auditors enhance the predictive ability of fair value measurements (Cantrell et al. 2014). Al-Hiyari et al. (2016) also favour Big 4 scrutiny, because the longer-term predictive ability of goodwill (second- and third-year-ahead cash flow from operations) seems to require a Big 4 auditor. However, in joint audits, Big 4-non-Big 4 auditor pairs compared to Big 4-Big 4 auditor pairs may enhance the predictive ability of goodwill (Lobo et al. 2017). Israeli (2015) provides further monitoring implications by showing with additional controls that external appraisers are usually positively related to the predictive ability while Big 4 auditors mostly yield insignificant results. Table 3 summarizes the main results regarding earnings persistence and predictive ability.

**Table 3:** Studies on earnings persistence and predictive ability

Year of publication	Author(s)	Region(s) Sample Period	Output variable(s)	Input variable(s)	Main result(s)	Moderating corporate governance
2019	Chung et al.	USA 576 observations 2006–2011	Cash flow from operating acitivities_t+1_ Operating Income_t+1_	= 1 if acquisition of in-process R&D at fair value	/ + (weak)	
2018	Black et al.	USA 3679 observations 2008–2014	ΔNet income	Fair valued assets Level 2 and 3*ΔNet income_t−1_ if variable is negative	–	Conservatism
2018	He et al.	Australia 237 observations 2001–2012	Net cash flows from operating activities_t+1_	Unrealized change in fair value of biological assets* = 1 if fair value Level 3	/	
2018	Yao et al.	Transnational (22 worldwide IFRS-applying countries) 871 observations 2009–2013	Earnings before taxes_t+1_	Fair valued financial assets Level 1 and aggregated*Earnings before taxes	+	Enforcement, audit environment, auditor industry expertise
				Fair valued financial assets Level 2 and Level 3*Earnings before taxes	/	
2017	Bandyopadhyay et al.	Canada 173–183 observations 2011–2014	Cumulative operating cash flow_t+2–4_	Fair value adjustment gain or loss for investment property	+	Conservatism
2017	Lobo et al.	France 527 observations 2006–2009	Operating cash flow_t+1_	Goodwill	+	Joint audit pairs
				Goodwill* = 1 if both Big 4 auditors	−	
2017	Shaari et al.	Malaysia 182–128 observations 2006–2009	(Δ)Net cash flow from operations_t+1_	Impairment reversal	/	
			(Δ)Operating income_t+1_	Impairment reversal*1 if non-earnings manager	+	
2016	Al-Hiyari et al.	Malaysia 726 observations 2011–2012	Cash flow from operations_t+2,3_	Goodwill	/	Big 4 auditor
2016	Bratten et al.	USA 6485–5566 observations 2001–2013	Earnings before taxes_t+1,2_	Pre-tax unrealized gains and losses from fair value changes of available-for-sale securities	+	
				Pre-tax unrealized gains and losses from fair value changes of derivatives classified as cash flow hedges	−	
2016	Xie	USA 34,212 observations 1994–2013	Unrealized gains and losses from available-for-sale securities	Fair value of available-for-sale securities_t−1_	−	
				Fair value of available-for-sale securities_t−1_* = 1 if recession period	/	
2015	Campbell	USA 4980 observations 2001–2006	ΔGross profit_t+2_	Unrealized hedging gains and losses in accumulated OCI	−	
2015	Filip et al.	USA 4451 observations 2003–2011	ΔEarnings before interests, taxes, depreciation, and amortization t_t+1_	Suspects of postponing goodwill impairment	+ (weaker)	
				Control firms	+	
2015	Israeli	Transnational (France, Germany, Spain, Italy) 254–343 observations 2005–2010	ΔCash flows from operations_t+1,2_	Fair value of investment properties	+	
			ΔNet rental income_t+1,2_	Investment property revaluation gains and losses	+	
2014	Cantrell et al.	USA 3801 observations 2005–2009	Chargeoffs_t+1_	Net historical cost of loans	−	Audit committee expertise, Big 4 auditors
				Fair value of loans	− (weaker)	
2014	Dong et al.	USA 973 observations 1998–2006	Comprehensive income_t+1_	Reclassified accumulated unrealized gains and losses from available-for-sale securities	+	
2014	Evans et al.	USA 7794 observations 1994–2007	Securities income_t+1_, Securities-related interest income_t+1_	Fair value—amortized cost for investment securities	+	
2013	Altamuro and Zhang	USA 382–978 observations 2008–2011	Mortgage servicing revenue_t+1_	Mortgage servicing revenue	+	
2013	Makar et al.	USA 708 observations 2001–2006	Cash flows from operations_t+1_	OCI adjustment for cash flow hedges	−	
2012	Lopes and Walker	Brazil 135 observations 1998–2004	ΔOperating income_t+1,2,3_	Net increment in revaluation reserve	−	Corporate governance index
2011	Goncharov and van Triest	Russia 4424 observations 2003–2006	Net income	Increment on revaluation of financial assets_t−1_	/	
				= 1 if upward revaluation of financial assets_t−1_	+	
2011	Lee	USA 13,853–14,202 observations 1996–1998, 2002–2004	Cash flow from operations_t+1_	= 1 in post-SFAS 142*Goodwill	+	
				= 1 in post-SFAS 142*Goodwill charge	+	
2009	Jarva	USA 234–327 observations 2002–2005	Operating cash flow_t+1,2_	Goodwill write-off	+	
2003	Beaver and Venkatachalam	USA 869 observations 1992–1995	Net income before loan loss provisions_t+1,2_	Discretionary and noise component of loan fair values	+	
1999	Aboody et al.	UK 597–737 observations 1983–1995	ΔOperating income_t+1,2,3_ ΔCash from operations_t+1,3_	Net increment from fixed asset revaluation	+	
1999	Park et al.	USA 455 observations 1993–1995	Earnings before discontinued operations and extraordinary items_t+1_	Fair value—historical cost value of available-for-sale securities	+	

The column ‘Main results’ states mostly positive (+), negative (−), or insignificant (/) associations between output and input variables

### Discretionary accruals

Discretionary accruals are positively associated with non-current asset revaluation (Hu et al. 2015), suspected goodwill impairment postponements (Filip et al. 2015), and fair valued investment properties (Hsu and Wu 2019), which indicates the opportunistic exploitation of fair value measurements. However, two studies find the opposite. Iatridis and Kilirgiotis (2012) show that fixed asset revaluation is negatively associated with high discretionary accruals, and they propose that the positive effects of fixed asset revaluation may reduce earnings management incentives. Additionally, Choi et al. (2015) investigate whether derivative hedging remains a tool for earnings management after mandatory recognition of transactions at fair value. The results show no substitution relation between discretionary accruals and fair valued derivative hedging, which suggests that managers use other tools instead of fair value measurements for earnings management (Choi et al. 2015).

Regarding moderating corporate governance, managers may fear auditors’ monitoring of fair value measurements (Filip et al. 2015). Big 4 auditors are found to decrease the association of fair value measurements and discretionary accruals that suggests less adverse earnings management (Hu et al. 2015), or not to influence this relation (Choi et al. 2015). Additionally, other corporate governance mechanisms are incorporated. While the SOX requirements of a majority of independent directors and an independent audit committee are unrelated (Choi et al. 2015), internal director revaluation increases discretionary accruals, and a self-constructed corporate governance index of board characteristics shows a decreasing effect (Hu et al. 2015). Table 4 summarizes the main results regarding discretionary accruals.

**Table 4:** Studies on discretionary accruals

Year of publication	Author(s)	Region(s) Sample Period	Output variable(s)	Input variable(s)	Main result(s)	Moderating corporate governance
2019	Hsu and Wu	China 2607 observations 2007–2011	Discretionary accruals	Investment property* = 1 if fair value model	+	
2015	Choi et al.	USA 811 observations 1996–2006	Discretionary accruals	Notional or fair market value of derivatives* = 1 if SFAS 133 (2002 onwards)	+	Big 4 auditor, director independence, audit committee independence
2015	Filip et al.	USA 4808 observations 2003–2011	Discretionary accruals	= 1 if suspected of goodwill impairment postponing	+	
2015	Hu et al.	Australia 951 observations 2003–2007	Discretionary accruals	= 1 if non-current asset revaluation	+	Big 4 auditor, board characteristics
2012	Iatridis and Kilirgiotis	UK 239 firms 2007	= 1 if high discretionary accruals	= 1 if fixed asset revaluation	−	

The column ‘Main results’ states mostly positive (+) or negative (−) associations between output and input variables

### Target beating and properties of analysts’ forecasts

FASB’s 2009 relaxation of fair value measurement application may increase discretion (Fargher and Zhang 2014). The authors show that an increase of fair valued assets of Level 2 and 3 in the post-relaxation period is positively associated with a slight beating of analysts’ earnings forecasts. Another unique setting to investigate discretion in fair value accounting is the early adoption period of SFAS 159. Firms may record differences between the carrying amounts and fair values of existing financial assets and liabilities in retained earnings at the balance sheet instead of recording them at the income statement (Guthrie et al. 2011). Henry (2009) provides confirming indications of adverse earnings management. However, Guthrie et al. (2011) fail to find opportunistic adoption, whether in the beating of earnings forecasts or in the realization of significant positive earnings (Guthrie et al. 2011). Managers of real estate investment funds seem to manage earnings via asset valuations so as not to report a decline in net asset values (Pinto 2013). Audit quality may mitigate this behavior, and financial distress may enhance it (Pinto 2013). Except for Guthrie et al. (2011), the results so far are consistent with adverse earnings management.

Other studies investigate the accuracy or dispersion of analysts’ forecasts instead of meeting or beating them slightly. Overall, fair value measurements and related disclosure may contribute to analysts’ forecasting ability under some conditions (Ayres et al. 2017; Liang and Riedl 2014; Paugam and Ramond 2015). This is in line with managers communicating private information to analysts via fair value measurements (Liang and Riedl 2014). However, fair value adjustments in the profit and loss statement appear to complicate earnings-per-share forecasts (Liang and Riedl 2014). Additionally, material write-offs are unrelated to analysts’ forecast accuracy and do not seem to surprise analysts, that is, they are incorporated by analysts (Jarva 2014). Fair values of Level 1 (Ayres et al. 2017) and Level 2 (Ayres et al. 2017; Magnan et al. 2015) seem to drive the association of fair value measurements and forecast accuracy. Fair value measurements of Level 3 are unrelated to forecast accuracy (Ayres et al. 2017; Magnan et al. 2015) and may even cause a dispersion of forecasts (Magnan et al. 2015). This is consistent with the assumption that higher-level fair value measurements enhance the quality of private (and public) information, while Level 3 fair value measurements reduce it (Ayres et al. 2017; Magnan et al. 2015). Besides the disclosure of levels, managers have an opportunity to guide analysts’ forecasts actively via the quality of the fair value-related disclosure. Providing only boilerplate information does not significantly influence analysts’ predictions, but the disclosure of relevant information seems to reduce their forecast error (Paugam and Ramond 2015). Table 5 summarizes the main results regarding target beating and properties of analysts’ forecasts.

**Table 5:** Studies on target beating and properties’ of analysts’ earnings forecasts

Year of publication	Author(s)	Region(s) Sample Period	Output variable(s)	Input variable(s)	Main result(s)	Moderating corporate governance
2017	Ayres et al.	USA 13,990 observations 2007–2013	Analysts’ earnings forecast accuracy	Fair value assets and liabilities	+	
2015	Magnan et al.	USA 649–5963 observations 1996–2009	Analysts’ earnings forecasts (accuracy/dispersion)	Fair value assets and liabilities	− (disp.)	
				Fair value assets and liabilities Level 2	+ (acc.)	
				Fair value assets and liabilities Level 3	− (disp.)	
2015	Paugam and Ramond	France 445 observations 2006–2009	Analysts’ earnings forecast error	Prospective impairment-testing disclosure score	−	
2014	Fargher and Zhang	USA 3431 observations 2007–2011	= 1 if analysts' earnings forecast error from 0 to 1 cent per share	= 1 if post-relaxation period of fair value rule (2009 onwards)* = 1 if increase in Level 2 and 3 fair valued assets	+	
2014	Jarva	USA 415 observations 2002–2005	Analysts’ earnings forecast accuracy	= 1 if material goodwill write-off	/	
2014	Liang and Riedl	Transnational (USA, UK) 754–828 observations 2002–2010	Analysts’ net asset value forecast error	= 1 if US-listed investment property firm that does not report property fair values	+	
			Earnings-per-share forecast error	= 1 if UK firm that reports property fair values* = 1 if post-IFRS	+	
2013	Pinto	Portugal 400 observations 2003–2009	Discretionary asset value changes of real estate investment funds in portfolios with immediately above zero net asset value returns		+	Big N auditor
2011	Guthrie et al.	USA 21 firms 2007–2008	Early adopters of fair value option with negative transition adjustments to retained earnings instead of income		52% (des.)	
2009	Henry	USA 35 firms 2007	Early adopters of fair value option with negative transition adjustments to retained earnings instead of income		94% (des.)	

The column ‘Main results’ states mostly positive (+), negative (−), or insignificant (/) associations between output and input variables. We also highlight specific descriptive (des.) evidence

### Earnings variability

Research indicates that fair value accounting (Barth et al. 1995; Hodder et al. 2006; Kohlbeck and Warfield 2010), and specifically mark-to-market accounting (Bernard et al. 1995), induces a greater variability of earnings than historical cost accounting. Therein are some indications that the lower discretionary earnings components of fair value accounting, or rather mark-to-market accounting, drive the variability (Barth et al. 1995; Bernard et al. 1995), which is inconclusive with earnings management.

The adoption of IFRS, and therefore IAS 39, seems to affect the earnings variability positively (Duh et al. 2012). The authors mainly attribute this to the increased use of fair value accounting. In an international IFRS sample, banks that apply the fair value option show lower earnings variability than other banks (Fiechter 2011). Applying the fair value option in response to accounting mismatches appears more common in countries with high regulatory quality (Fiechter 2011). Managers in these countries seem to use the fair value option as intended, which enhances earnings quality. However, in a U.S. sample, financial institutions that apply the fair value option show increased earnings volatility (Couch et al. 2017). This association appears stronger for firms that only use the fair value for assets and not for liabilities, and they provide indications that Level 3 fair value measurements contribute to this decision (Couch et al. 2017). Taken together with a less restrictive fair value option under US-GAAP (Couch et al. 2017), managers may not act as intended and exploit greater discretion under US-GAAP compared to IFRS for opportunistic purposes. Table 6 summarizes the main results regarding earnings variability.

**Table 6:** Studies on earnings variability

Year of publication	Author(s)	Region(s) Sample Period	Output variable(s)	Input variable(s)	Main result(s)	Moderating corporate governance
2017	Couch et al.	USA 360–556 firms 2004–2007, 2009–2012	σ(Operating income)	= 1 if Adoption of fair value option	+	
2012	Duh et al.	Transnational (14 countries) 277 observations 2000–2009	σ(Net income) σ(Comprehensive income)	= 1 for years 2005 onwards* = 1 if country mandatorily adopt IFRS from 2005 onwards	+	
2011	Fiechter	Transnational (41 countries) 207–222 firms 2006–2007	σ(Earnings before taxes)	= 1 if fair value option primarily applied under eligibility criterion 1 (accounting mismatch)	−	Regulatory quality
2010	Kohlbeck and Warfield	USA 91,931 observations 1976–2005	Increase of variance of residuals from estimation	SFAS 115	105% (des.)	
				SFAS 133	245% (des.)	
2006	Hodder et al.	USA 202 firms 1996–2004	σ(Full fair-value income) exceeds	σ(Net income)	455% (des.)	
				σ(Comprehensive income)	266% (des.)	
1995	Barth et al.	USA 1859 observations 1971–1990	σ(Fair value net income) exceeds	σ(Historical cost net income)	+	
				σ(Net income before securities gains and losses)	+	
1995	Bernard et al.	Denmark 57 firms 1976–1989	σ(mark-to-market accounting adjustments) exceeds	σ(Earnings before loan loss provisions, price adjustments, depreciation, extraordinary items, and taxes)	27–284% (des.)	

The column ‘Main results’ states mostly positive (+), or negative (−) associations between output and input variables. We also highlight specific descriptive (des.) evidence

### Other earnings quality measures

Accounting restatements are another indicator of (decreased) earnings quality (Hribar and Jenkins 2004; Lin et al. 2017). Overall, Level 3 fair valued assets are positively related to accounting restatements, indicating lower earnings quality (Lin et al. 2017).^8^ They provide indications that the proposed effect mitigates with stronger corporate governance quality, based on an index that comprises board independence, financial expertise, size of audit committee, share of institutional investors, size of audit office, and no material control weaknesses (Lin et al. 2017). Similar to accounting restatements, Bens et al. (2016) investigate SEC comment letters on fair value accounting as enforcement activities. They find that the all-level fair valued assets are negatively related to receiving a SEC comment letter on fair value accounting, while the fair valued liabilities and an indicator for large amounts of Level 3 fair valued assets are positively related.

Another approach to investigating earnings quality is aggregating different earnings quality measures. Tutino and Pompili (2018) aggregate the accounting-based measures’ predictability, persistence, and volatility, along with the market-based measures’ value relevance and conservatism. The authors interpret a positive association of fair value exposure with their aggregated measure as lower earnings quality for U.S. banks, while they find no relation for European banks. Table 7 summarizes the main results.

**Table 7:** Studies on other earnings quality measures

Year of publication	Author(s)	Region(s) Sample Period	Output variable(s)	Input variable(s)	Main result(s)	Moderating corporate governance
2018	Tutino and Pompili	Transnational (USA, EU) 132 firms 2011–2016	Aggregate (low) earnings quality measure	ΔFair valued assets and liabilities through net income	+ (USA) / (Europe)	
2017	Lin et al.	USA 10,104 observations 2008–2010	= 1 if restatement after Level 3 fair value disclosure	Level 3 fair values of financial assets	+	Corporate governance index
2016	Bens et al.	USA 6760 observations 2007–2012	= 1 if receiving a fair value comment letter	Fair valued assets	−	
				Fair valued liabilities	+	
				= 1 if higher proportion of Level 3 fair value assets	+	

The column ‘Main results’ states mostly positive (+), negative (−), or insignificant (/) associations between output and input variables

## Limitations and recommendations for future research

At first glance, the results of the reviewed studies appear mixed. In this section, we provide further insights by showing the limitations of the reviewed studies, which are widely based on the neoclassical principal-agent theory. We identify two key extensions of future research as limitations of the reviewed studies: (1) extending methodology and settings, and (2) extending corporate governance issues with regard to behavioral biases. These extensions offer the development of fair value accounting for practitioners and regulators, and provide further research questions (RQ). Thereby, we encourage future research to recognize each issue in the general context of our recommendations.

### Extending future research in methodology and settings

#### Methodology of accounting-based earnings quality research

##### General issues for improving earnings quality research designs

Common earnings quality research show a great research body but some major challenges remain (Dechow et al. 2010a). Related to these challenges, we highlight issues of internal and construct validity in research designs of reviewed studies, especially regarding assigning inferences to managerial discretion. Internal validity relates to the research design in general, i.e. whether the relation is causal or whether confounding effects can be excluded (Drost 2011). Therefore, we recommend addressing specific endogeneity issues. Construct validity relates to whether the research designs operationalize theory adequately (Trochim 2020).^9^ We try to provide some practical guidance on how to increase construct validity via improving accounting-based earnings quality research designs. This is especially crucial for fair value accounting, because the body of common earnings quality research is developed under historical cost accounting (DeFond 2010).

Translation validity requires intuitive plausibility (face validity) and comprehensive verification of operationalization (content validity) (Trochim 2020). While applied earnings quality proxies appear plausible, research designs hardly enable inferences on whether the observed association can be related to managerial decisions or to circumstances beyond managerial sphere of influence. One example is the extent discussion of whether discretionary accruals approximate earnings management appropriately (Jackson 2018; Dechow et al. 2010a; DeFond 2010). Translating managerial discretion into the research designs may be strengthened by two means. First, researchers may exploit managerial incentives because they influence earnings quality proxies (Dechow et al. 2010a). Jarva (2009), Lee (2011), Filip et al. (2015), and Shaari et al. (2017) provide initial indications by incorporating earnings management suspects in their research designs. Other studies try to add robustness against managerial incentives and consider ratios of balance sheet and income statement items, like debt ratios or interest coverage ratio (e.g. Israeli 2015; Yao et al. 2018). Future research may incorporate incentives for earnings management to greater extent and more precise by hand-collecting data directly from annual reports, like closeness to covenants. Researchers may also obtain specific fair value-related pro-forma measures, such as changes to EPRA Net Asset Value metrics in the real estate industry (EPRA 2019). Second, textual analyses complement archival- and accounting-based earnings quality research in many ways, especially in sectors with extensive fair value disclosure, such as real estate firms or financial institutions. Investigating these disclosure practices whether managers want to convey or conceal information may help researchers to evaluate recognized items (Teoh 2018). Additionally, the style of qualitative information also enables the investigation of behavioral biases (Li 2010), which we introduce in the back of this section regarding fair value measurements. Furthermore, future research might investigate the readability of disclosed information that is also associated with earnings quality (Li 2008; Loughran and McDonald 2014). It can be measured via the size of information (Loughran and McDonald 2014). Thereby, future research can easily apply it in fair value-intensive sectors.

Criterion-related validity can be strengthened by verifying the operationalization with (external) references (Trochim 2020; Drost 2011). Studies already add convergent validity by providing variation in applied proxies (e.g. Filip et al. 2015; Magnan et al. 2015; Aboody et al. 1999). We suggest that future studies also increase predictive validity. If earnings quality research designs adequately indicate earnings management, suspected firms may be identified as earnings manager retrospectively. Ex post analyses of public noncompliant behavior provide another mean to investigate earnings management and may add robustness to other proxies (Jackson 2018). Among reviewed studies, Bens et al. (2016) and Lin et al. (2017) already provide ex post analyses by investigating SEC comment letters and accounting restatements. Hand-collection of ex post cases may provide further research opportunities. Thereby, future research designs may either collect indications of fair value or non-fair value specific noncompliant behavior from firm disclosures, information of regulators and enforcement authorities, or press releases regarding earnings management.

Besides different construct validity issues, we suggest that experiments complement archival- and accounting-based earnings quality research to support evidence of earnings management. Controlling and manipulating experimental settings permit direct inferences of managerial earnings management behavior in connection with fair value accounting (e.g. Mazza et al. 2011; Chen et al. 2013; Trottier 2013). This also strengthen internal validity. Furthermore, experiments provide detailed insights in the relevance of different fair value measurements and disclosure, whereby these regimes do net need to be amended (Koonce et al. 2011; Lachmann et al. 2015; Clor-Proell et al. 2014). Consequently, experimental evidence will provide further insights in managerial behavior and whether they are successful in managing earnings.

RQ1: Do previous results change if fair value-related earnings quality research designs are modified and supplemented using additional managerial incentives, textual analyses, ex post analyses, and experiments? Do a mixture of different research design issues add further validity to the relation between fair value measurements and earnings quality?

##### Specific issues for mitigating endogeneity issues

In line with the broader accounting, finance, and corporate governance literature (Wintoki et al. 2012; Brown et al. 2011; Armstrong et al. 2010), endogeneity issues in fair value accounting research, and especially in investigating fair value measurements’ earnings quality, arise from a lack of sufficient exogenous settings. Reviewed studies use different techniques to add additional robustness to their studies by variations in their research designs. For example, Cantrell et al. (2014), Evans et al. (2014), and He et al. (2018) provide out-of-sample tests. The majority of studies provide variations in the output and input variables, like some earnings persistence and predictive ability studies provide variation of time-horizon for calculating the output variable (e.g. Bandyopadhyay et al. 2017) or use cash flows instead of earnings (e.g. Chung et al. 2019). Among reviewed studies, selection biases may be the most concerned specific endogeneity issue. Researchers apply different techniques to their accounting-based earnings quality designs to tackle these issues or at least to add additional robustness to their models. Reviewed studies use treatment effects models or selection models (e.g. Heckman 1979) to address selection biases regarding business decisions to use specific items (Makar et al. 2013; Campbell 2015) or reporting decisions of specific items (Israeli 2015). These techniques are also used to account for applied reporting regimes (Couch et al. 2017; Fiechter 2011) or different firm characteristics (Bandyopadhyay et al. 2017; Jarva 2009). Additionally, reviewed studies apply matching procedures (e.g. Shipman et al. 2017) to control for (non-)impairment decisions (Filip et al. 2015; Jarva 2014, 2009), for firms that (do not) report specific information (Lin et al. 2017; Altamuro and Zhang 2013), or for applied reporting regimes (Fiechter 2011).

We emphasize that there might be another endogeneity issue within fair value accounting research, that is, reverse causality. Some studies indicate that earnings quality measures, such as earnings variability (Quagli and Avallone 2010), specific accruals (Chong et al. 2012), and discretionary working capital accruals (Cao et al. 2018) are determinants of fair value measurements or whether they are applied. Consequently, the causality of the relation between fair value measurements and earnings quality measures is unclear: either fair value measurements influence earnings quality or earnings quality influences fair value measurements. Future research might apply two means to tackle reverse causality and other endogeneity issues.

First, sound theoretical reasoning is a basic and important instrument to mitigate endogeneity concerns (Chenhall and Moers 2007; Larcker and Rusticus 2007; Gassen 2014). However, some reviewed studies lack in coherent and comprehensive theory. For example, we observe a twofold and diverging theoretical foundation with different assumptions regarding the relation between fair value measurements and discretionary accruals: On the one hand, the authors interpret a positive association of fair value measurements and discretionary accruals as increased fair value-based earnings management (Hu et al. 2015; Filip et al. 2015). The underlying assumption is that fair value measurements drive discretionary accruals (complementary view). On the other hand, Choi et al. (2015) would interpret a negative association as increased fair value-based earnings management (substitutional view). The underlying assumption here is that firms engage either in fair valued transactions (hedging in this case) or in managing accruals for earnings management purposes. This assumption is more related to real earnings management (Barton 2001; Pincus and Rajgopal 2002; Kilic et al. 2013). We suggest that future research need to discuss different assumptions and theoretically rule out more than one potential interpretation of the investigated relation to mitigate endogeneity issues.

Second, natural quasi-experimental settings provide unique opportunities to further mitigate endogeneity concerns in general (Gassen 2014; Gippel et al. 2015). More specifically, exploiting these settings help to mitigate concerns regarding initially stated selection biases (Shipman et al. 2017). Most of the reviewed studies investigate the adoption of newly issued standards but fewer studies exploit the discontinuity in reporting pre- and post-adoption (e.g. Lee 2011; Couch et al. 2017; Chung et al. 2019). However, future studies might exploit regime changes to a greater extent. Therefore, we mention ideas for specific regulatory shocks to fair value regimes and governmental structures as well as macroeconomic shocks in the next section.

RQ2: Do more sophisticated settings as well as more theoretical analysis mitigate endogeneity concerns and add further validity to the relation between fair value measurements and earnings quality?

#### Fair value measurement settings

##### External shocks as unique settings

Current and recent developments offer unique settings that may be exploited for further investigation of the relation between fair value measurements and earnings quality, which help to mitigate endogeneity concerns. First, macroeconomic shocks provide unique settings for investigating managerial discretion. Some reviewed studies investigate whether the financial crisis affects the relation between fair value measurements and earnings quality (Liang and Riedl 2014; Bratten et al. 2016; Xie 2016). More recently, the coronavirus pandemic needs to be incorporated. It creates general uncertainties with great potential to affect fair value measurements (IAASB 2020). One specific example is the real estate industry. According to experts Warren Buffett and Barbara Corcoran, the coronavirus pandemic can change office space renting business and decrease renting demand in the long run (Paynter 2020a, b). This decline would decrease expected cash flows that require adjustments to fair value measurements of these business units.

Second, regulatory shocks to governmental structures create unique settings. EU directive 2017/828 introduces new corporate governance regulations to the European Union to strengthen investors commitment in the long run. Since fair value-based earnings management reverse in future periods, long run engagement and associated investor oversight may alter this behavior. Other national regulation can also affect firms’ way of doing business, their cash flows, and finally their fair value measurements. For example, real estate firms are concerned by additional regulation due to public pressure as a response of rising housing prices like in Germany (Vonovia SE 2020; Deutsche Wohnen SE 2020).

Third, regulatory shocks to fair value regimes create unique settings. A research opportunity may be the expected loss model of IFRS 9 for determining impairment losses (IFRS 9.5.5), which enables and demands greater managerial discretion in considering issues of future periods (Lobo 2017). It replaces the less discretionary (Gebhardt and Novotny-Farkas 2011) incurred loss model (IAS 39.58-70 superseded). Another example is the IFRS adoption in Brazil. During this process (2007–2010), asset revaluation was abolished although previous local GAAP included asset revaluation (Lopes and Walker 2012). The authors also mention that asset revaluation is usually permitted after IFRS adoption in other countries while it was not allowed beforehand.

RQ3: Does the exploitation of macroeconomic shocks as well as regulatory shocks to governance structures and fair value regimes provide additional evidence regarding the relation between fair value measurements and earnings quality?

##### Investment properties as a specific suitable setting

Although we admit that 48 reviewed studies make up only a small proportion of research, we highlight certain characteristics of the applied research settings that may guide future research. Despite our observation that studies applying IFRS or similar standards settings (IFRS settings) find relatively more earnings quality enhancing effects of fair value measurements than studies of solely US-GAAP appliers (US-GAAP settings), IFRS settings are underrepresented among the reviewed studies. However, the trend of dominating US-GAAP settings is slightly decreasing from about 58% among all reviewed studies to 42% among reviewed studies that are published in 2017, 2018, and 2019. Moreover, financial industry-related settings or settings that obtain fair value measurements from financial items (financial settings), which make up 56% of reviewed studies, outnumber non-financial settings. Studying these patterns, we observe that investigating financial settings appear prominent among US-GAAP appliers, et vice versa. Both characteristics, financial settings and US-GAAP settings, show a correlation coefficient of 0.4.^10^ Therefore, we recommend conducting more research among non-financial IFRS settings. As a suitable example, we highlight a certain setting in detail, that is, investment properties.

Besides providing the opportunity of fair value research in a non-financial and non-US-GAAP setting, investigating investment properties has further advantages: first, fair value measurements of investment properties are usually based on discretionary lower-level inputs (PricewaterhouseCoopers LLP 2017; Goncharov et al. 2014; Dietrich et al. 2001). About a quarter of reviewed studies investigate levels of fair value measurements and most of them find that lower-level (Level 3 and Level 2, or just Level 3) fair value measurements may decrease earnings quality.^11^ Second, the subjected assets account for material shares of real estate firms’ balance sheets on average (Israeli 2015). Third, adjustments to fair values of investment properties are shown in profit or loss (IAS 40.35) and not in OCI, like several other fair value adjustments. Fourth, accounting-based earnings quality research of fair valued investment properties is far from being conclusive. Previous studies provide mixed results despite a slightly earnings quality enhancing effect: fair value measurements according to IAS 40 or similar standards may enhance earnings quality (Bandyopadhyay et al. 2017; Israeli 2015; Liang and Riedl 2014). However, results from China (Hsu and Wu 2019) and results of the specialized research design of Pinto (2013) indicate that fair value accounting of investment properties decreases earnings quality. Additional results of the related revaluation model appear even more mixed.

RQ4: Do fair value measurements of fair valued investment properties enhance earnings quality? Do fair valued investment properties provide different results than other lower-level fair value measurements?

### Extending future research with (behavioral) corporate governance issues

According to traditional agency theory, stronger corporate governance faces lower agency problems (Shleifer and Vishny 1997). About a quarter of the reviewed studies apply moderating corporate governance mechanisms. Nearly all studies show that (predicted stronger) corporate governance quality tends to enhance the influence of fair value measurements on earnings quality. Because this enhancing effect is incorporated only by a quarter of the reviewed studies, we see a research gap in the further application of moderating corporate governance.

The third, group level corporate governance mechanisms include board characteristics and CEO compensation^12^ (Jain and Jamali 2016). Boards and managerial compensation are material determinants of earnings management (Laux and Laux 2009). Board characteristics and especially audit committee characteristics, i.e. expertise, seem to enhance the earnings quality of fair value measurements (Cantrell et al. 2014; Lin et al. 2017) or do not influence this relation (Choi et al. 2015). However, reviewed studies neglect managerial compensation.

CEO compensation may either influence accruals management (Bergstresser and Philippon 2006) and meeting or beating analysts’ forecasts (Cheng and Warfield 2005). Thereby, we perceive CEO compensation is a substantial driver for fair value-related earnings management. On the one hand, it may also influence managerial earnings forecasts (Kim et al. 2019), which are fundamental for estimating Level 3 fair value measurements. On the other hand, fair value measurements are already found positively related to CEO compensation, especially to cash bonuses (Livne et al. 2011). Accounting-based compensation measures are perceived to remain unadjusted for unrealized gains and losses from fair value accounting (Livne and Markarian 2018), which may encourage managers to exploit fair valued items opportunistically (Shalev et al. 2013). Future research might incorporate several corporate governance mechanisms to account for CEO compensation. First, financial knowledge in the compensation committee is expected to enhance the adequacy of compensation contracts (Manchiraju et al. 2016). Therefore, we suggest secondarily that both audit committees, as introduced among the reviewed studies, and compensation committees appear essential for investigating fair value-related earnings management. The effects of director overlap between both committees are either complex (Laux and Laux 2009) but can enhance earnings quality (Chandar et al. 2012). Third, compensation relevant unrealized gains and losses of fair value measurements particularly induce claw-back problems in case of no materialisation in later periods (Livne et al. 2011). Clawback provisions are found to enhance earnings quality in general (Chan et al. 2012; Dehaan et al. 2013; Natarajan and Zheng 2019) and help to mitigate this problem specifically. They are expected to constrain managerial behavior ex ante (Iskandar-Datta and Jia 2013) that may be of particular importance for fair value measurements because there are also doubts that boards are always capable to monitor fair value accounting properly (Dechow et al. 2010b).

RQ5: How can firms design corporate governance, especially compensation and board composition structures, to mitigate earnings management (incentives) via fair value measurements?

Researchers exploit circumstances around CEO compensation to construct proxies for CEO attributes, such as overconfidence (Malmendier and Tate 2005; Schrand and Zechman 2012; Arena et al. 2018). This and other CEO attributes are part of the fourth, individual level corporate governance mechanisms (Jain and Jamali 2016). Different individual CEO characteristics and behavior patterns are found to affect business decisions and financial reporting choices (Malmendier and Tate 2005; Capalbo et al. 2018). CEOs are threatened by overestimating chances, underestimating risks (overoptimism), and overestimating their abilities (overconfidence) (Heaton 2002; Malmendier and Tate 2005; Weinstein 1980). Excessive expression of these behavioral biases, in connection with the behavioral patterns of an unwillingness to update one’s beliefs and a strong will to maintain these biases in self-perception and outside perception, is described as narcissism (Post 1993; Rijsenbilt and Commandeur 2013). Previous authors also suggest that overoptimism and narcissism lead to adverse earnings management and lower earnings quality (Amernic and Craig 2010; Capalbo et al. 2018; Schrand and Zechman 2012). Because of fair value measurements’ exposure to subjectivity (Marra 2016), we expect that especially Level 3 fair value measurements are affected by these biases, for three reasons: first, because overoptimism and overconfidence induce overestimation in managerial forecasting (Heaton 2002; Hribar and Yang 2016), overoptimistic cash flow forecasts lead to overestimated Level 3 fair value measurements. Second, the narcissistic maintaining of unrealistic perceptions and the narcissistic inability to account for worsening conditions may even increase overestimation of Level 3 fair value measurements. Third, managers may seek to influence benchmarks for narcissistic perceptions, such as CEO compensation (O’Reilly et al. 2014) or positive reactions to financial reporting information (Amernic and Craig 2010), via managing fair value measurements.

RQ6: Do CEO overconfidence and narcissism influence the relation between fair value measurements and earnings quality?

## Conclusion

Earnings management via fair value measurements can either be used to convey information or be exploited opportunistically (Landsman 2007; Barth 2018; Yao et al. 2018), which may be mitigated by corporate governance (Shleifer and Vishny 1997). Earnings management via fair value measurements still remains an important and unsolved question for researchers and regulators because of its effect on the decision usefulness (Barth 2018; IASB 2018), and for practitioners that are interested in an unbiased contribution to performance evaluation (Georgiou 2018). Therefore, research, regulators, and practitioners need a comprehensive overview of whether managers use fair value measurements for earnings management purposes and how corporate governance affects this relation. Within this structured literature review, we focus on whether fair value measurements influence accounting-based earnings quality measures and how corporate governance variables moderate the aforementioned relation. We assume that accounting-based earnings quality measures enable more direct evidence of fair value-related accruals management (Francis et al. 2004; Bernard 1993; Aboody et al. 1999), whose interpretation do not require additional assumptions (Dechow and Schrand 2004; Dechow et al. 2010a), and provide more homogenous results than incorporating market-based earnings quality measures.

Thereby, we contribute to the previous literature in three ways. First, previous reviews incorporate or exclusively use market-based earning quality measures, such as value relevance studies (Barth et al. 2001; Holthausen and Watts 2001; Hairston and Brooks 2019). Second, we do not concentrate on specific industries or periods, unlike the previous reviews that often investigate financial industries and the financial crisis (e.g. Beatty and Liao 2014; Laux 2012). Third, we include all kinds of fair valued items. Some previous reviews investigate specific issues or items that are related to fair value accounting (e.g. Sellhorn and Stier 2019). Our final sample consists of 48 archival-based empirical studies. We classify reviewed articles according to the following categories: (1) earnings persistence and predictive ability, (2) discretionary accruals, (3) target beating and properties of analysts’ earnings forecasts, (4) earnings variability, and (5) other earnings quality measures.

We provide three common findings and further evidence, which we discuss as limitations and recommendations for future research. First, fair value measurements show mixed earnings quality. Second, lower-level fair value measurements decrease earnings quality, and third, stronger corporate governance enhances earnings quality. We partition six research questions that would extend future research regarding (1) methodology and settings, and (2) (behavioral) corporate governance issues. First, we recommend extending fair value-related earnings quality research designs with managerial incentives, textual analyses, ex post analyses, and experiments. Future research should also account for endogeneity concerns by providing more sound theoretical reasoning and exploiting unique settings. Examples for the letter are current and recent shocks to macroeconomic conditions as well as regulatory shocks to governmental structures and fair value regimes. As a specific setting, we recommend more accounting-based earnings quality research regarding investment properties. Second, we propose further incorporating of corporate governance mechanisms, especially regarding compensation and board structures. Future research should also consider behavioral biases.

The systematic structure of studies, summarized empirical findings, and discussed limitations and research questions help regulators to evaluate managerial discretion in fair value accounting on a more reasonable basis, which was left as challenging after the post-implementation review of IFRS 13 (IASB 2018). Furthermore, practitioners can use this evidence, especially regarding (behavioral) corporate governance, to align managerial behavior. On top of that, mentioned aspects should guide future research because we show which investigations regarding earnings quality of fair value measurements are most urgent and promise significant contributions.

## Footnotes

1 We refer to the common term *reliability*. We notice that the IFRS Conceptual Framework issued in 2010 and the revised Conceptual Framework issued in 2018 do not yield reliability as a separate fundamental qualitative characteristic as in the Framework adopted by the IASB in 2001; see also Sellhorn and Stier (2019).

2 In short, we proceed as follows: first, we define keywords of the intended topic and apply them to selected databases. We select archival-based articles of high scientific quality regarding the intended topic and build an empirical framework upon them. After summarizing the relevant literature, we provide the limitations and recommendations for future research.

3 According to the journal rankings, journals from the following categories are included: 4*, 4, 3, 2 (ABS Guide 2018), A*, A, B (ABDC 2016), and A+, A, B, C (VHB JOURQUAL 3).

4 We use the term ‘earnings quality’ and not ‘financial reporting quality’ because we do not discuss disclosure quality explicitly, although we refer to disclosed amounts where appropriate.

5 Dechow et al. (2010a) set up a third category of earnings quality proxies (*external indicators of earnings misstatements*) that cover restatements and enforcement activities, among others. Since these measures play a minor role in this review, we review them in Sect. 4.5 and stick to the term *accounting-based* measures.

6 The interpretation regarding smoothness is discussed in connection with the quality of accruals, which may require additional measurements. For reasons of brevity, we leave out further methodical discussions at this point.

7 Evans et al. (2014) do not separate between the classifications available-for-sale and held-to-maturity, but they admit that the share of held-to-maturity securities is substantially smaller than that of available-for-sale securities.

8 For restating financial statements according to US-GAAP, see ASC 250.

9 We rely on a well-recognized work of Trochim (2020) (more than 11,000 citations on Google Scholar on 23rd October 2020) to break down construct validity. It may be assessed via translation validity, which subsumes face and content validity, and criterion-related validity, which subsumes predictive, concurrent, convergent, and discriminant validity.

10 We calculate the correlation coefficient based on two binary variables that equal one if the reviewed studies apply a US-GAAP setting and if the reviewed studies apply a financial setting, respectively, and zero otherwise.

11 Therein, we also include evidence if lower-level fair value measurements do not enhance earnings quality, while aggregate fair value measurements do, or if lower-level fair value measurements enhance earnings quality less strongly than aggregate fair value measurements.

12 Note that compensation research mostly relates to the CEO.
